# Gallbladder agenesis in the elderly: a diagnostic challenge

**DOI:** 10.11604/pamj.2020.37.259.23268

**Published:** 2020-11-23

**Authors:** Imen Ben Ismail, Saber Rebii, Hakim Zenaidi, Ayoub Zoghlami

**Affiliations:** 1 Department of General Surgery, University of Tunis El Manar, Traumatology and Severe Burns Centre, Ben Arous, Tunisia

**Keywords:** Gallbladder agenesis, ultrasound, magnetic resonance cholangiopancreatography, case report

## Abstract

Gallbladder agenesis (GA) is a rare congenital malformation characterized by the absence of the gallbladder and cystic duct due to an anomaly in the embryonic development. It is commonly associated with other congenital abnormalities, and the isolated form is extremely rare. Its clinical presentation is variable. Actually, GA is more often incidentally diagnosed. Magnetic resonance cholangiopancreatography (MRCP) is considered to be the diagnosis method of choice since it avoids unnecessary and risky surgery in symptomatic patients. Here we report the case of a radiologically incidentally discovered gallbladder agenesis in a 68-year-old patient.

## Introduction

Gallbladder agenesis (GA) is a rare congenital malformation of the biliary tract characterized by the absence of the gallbladder and cystic duct [1]. It was first reported by Lemery in 1701 [2]. Pathogenesis of this anatomical abnormality can be explained by either complete lack of bud formation or lack of recanalization of the bud during its growth [1]. As it results to an anomaly in the embryonic development, gallbladder agenesis may be associated with other biliary congenital malformations and/ or extra-biliary, including gastrointestinal, genitourinary, musculoskeletal or cardiovascular systems [3]. The isolated agenesis of the gallbladder is extremely rare with a reported incidence of 0.01-0.06% [1]. It commonly affects females (ratio 3: 1) in the second and third decades of life [4]. Most affected patients are asymptomatic; furthermore, 50% of them may develop typical symptoms of gallbladder pathology such as dyspepsia, right upper quadrant pain, nausea, vomiting and jaundice [1], so that gallbladder agenesis will be misdiagnosed as cholecystitis leading to, unnecessary and risky surgery. The preoperative diagnosis of this rare entity is challenging, and MRCP is the diagnosis method of choice. We herein report the case of a radiologically incidentally discovered gallbladder agenesis in a 68-year-old patient.

## Patient and observation

A 68-year-old man, without any past medical or surgical history, presented to general surgery clinic for an uncomplicated umbilical hernia evolving for five years.

**Clinical findings**: physical examination showed an umbilical mass measuring about 4 cm. It was painless and utterly reducible with an expansile cough impulse. Otherwise, the abdomen was soft, with no guarding or tenderness.

**Timeline**: as part of the preoperative check-up, an abdominal ultrasound was performed. The latter did not visualize the gallbladder clearly, and this suggested the presence of a sclerotic or atrophied gallbladder. The biliary tree was normal, however. Reinterviewing the patient, he did not have any history of right upper quadrant pain or episodes of fever and jaundice.

**Figure 1:**
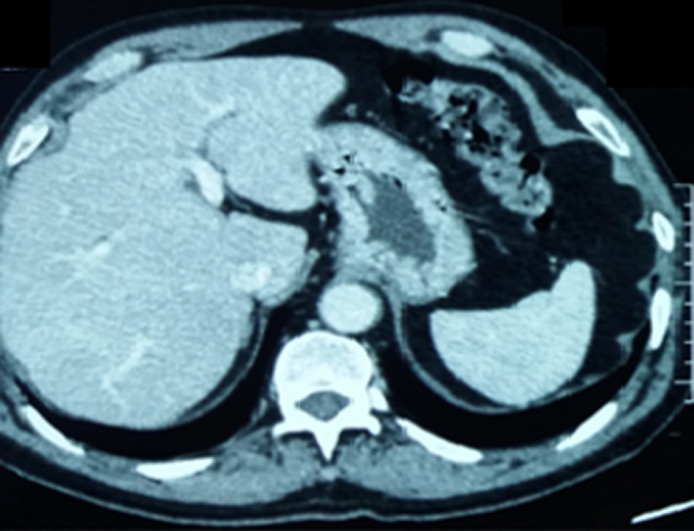
axial computed tomography scan of the hepatobiliary system revealing absence of the gallbladder in the cholecystic fossa

**Figure 2:**
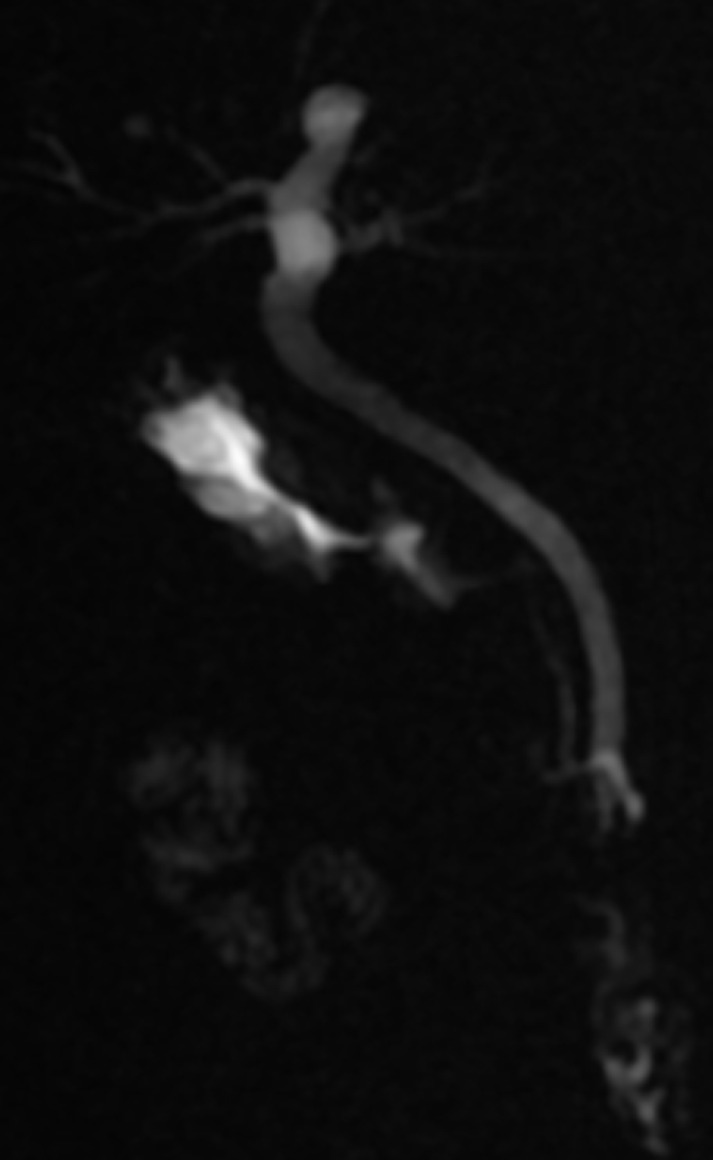
MRCP confirming agenesis of the gallbladder

**Figure 3:**
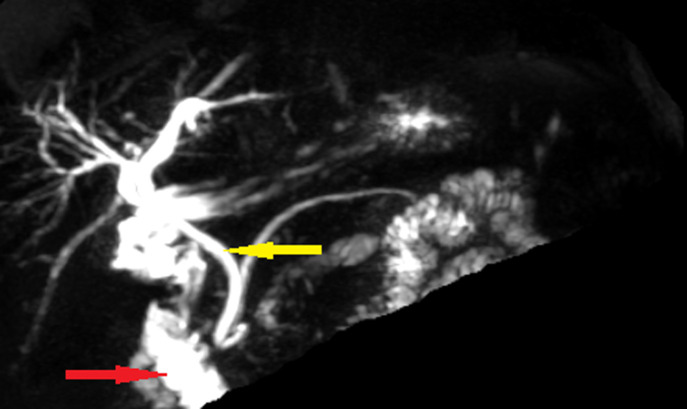
MRCP showing opacification of the entire biliary tract (CBD: yellow arrow) and duodenal framework (red arrow) without visualization of the cystic duct or gallbladder

**Diagnostic assessment**: biological findings were within normal limits; notably, liver function tests were unremarkable. A further investigation with an abdominal computed tomography (CT) scan was performed but failed to identify the gallbladder (Figure 1). Furthermore, there were no abnormalities detected in the biliary tract and the common bile duct measured 5 mm in diameter. Given non-visualization of the gallbladder on both ultrasound and CT scan, an MRCP was subsequently performed, revealing the absence of both the gallbladder and cystic duct (Figure 2, Figure 3). Then MRCP confirmed the diagnosis of gallbladder agenesis and ruled out an ectopic gallbladder.

**Therapeutic intervention**: since the patient was asymptomatic, no further management was required. He was then operated on for his umbilical hernia.

**Follow-up and outcomes**: the postoperative course was uneventful, and the patient remained symptom-free during a total follow up of two years.

## Discussion

Gallbladder agenesis is a rare congenital anomaly that can be observed in both children and adults. In general population, its estimated incidence ranges from 10 to 65 per 100,000 [1]. In autopsy series, the incidence is slightly higher (0.04-0.13%) [5]. Anatomically, the gallbladder develops from the caudal part of the hepatic diverticulum in the fourth week of embryonic life. Two theories can explain the agenesis of the gallbladder: the first theory suggests that the hepatic diverticular bud of the foregut fails to develop properly into the gallbladder and cystic duct. The second suggests that, following solid-phase development, there is a failure of recanalization of the cystic duct and gallbladder [1]. In 12.8 to 30% of cases, gallbladder agenesis is associated with other congenital abnormalities, mainly cardiovascular, gastrointestinal and genitourinary ones, such as ventricular septal defect, imperforate anus, duodenal atresia, malrotation of the gut, pancreas divisum, hypoplasia of the right hepatic lobe, duplication cysts of the hepatic flexure, renal agenesis, undescended testes, and syndactyly [6]. Among these associated malformations, anomalies of the bile tree, like atresia of the bile ducts and choledochal cyst are found in 9% of cases [7]. Clinically, gallbladder agenesis has no characteristic symptomatology. Bennion *et al*. [1], classified patients in three groups: the first group of asymptomatic patients where the diagnosis of GA is incidentally made at autopsy or surgery for other reason (35%). The second group of symptomatic ones (50%), and the third one of children with other severe congenital anomalies/6. In our case, the patient was asymptomatic, and the diagnosis was made incidentally at routine ultrasonography performed as part of a preoperative check-up for umbilical hernia repair. In symptomatic cases, GA commonly manifests itself with right upper quadrant abdominal pain (90%), nausea and vomiting (66%), fatty food intolerance (37%), dyspepsia (30%), and jaundice (35%) [8]. Pathophysiologically, these symptoms can be explained with stasis in the bile duct, which elevates the basal pressure of the Oddi sphincter generating biliary colic [9]. Others think that choledocholithiasis secondary to biliary dyskinesia may be the leading cause of these symptoms [10]. Previously, the diagnosis of GA was challenging and commonly made at surgery. Nowadays, thanks to the development of imaging modalities, the diagnosis can be made preoperatively, and unnecessary surgery can be avoided.

If the diagnosis is made during surgery, the surgeon must rule out an ectopic gallbladder by examining the most common sites for ectopic gallbladder, which are intrahepatic, retrohepatic, on the left side, or within the leaves of the lesser omentum or within the falciform ligament, retroduodenal, retropancreatic, and retroperitoneal [11]. This meticulous surgical exploration heightens the risk of biliary injury and increases the need to convert to open access. That is why it is recommended to abort the procedure rather than complete further exploration if the gallbladder is not found on laparoscopy and final diagnosis would be confirmed by a postoperative careful radiologic investigation [12]. Ultrasonography is highly sensitive (95%) imaging modality for gallbladder diseases, but it depends hugely on the operator´s experience and the examination conditions. In our case, the ultrasound was misleading. Similarly, most of the reported cases of GA were diagnosed as a “contracted/fibrotic gallbladder” on ultrasound [12]. CT scan is not very helpful too since non-visualization of the gallbladder can be due to cystic duct obstruction or gallbladder agenesis [13]. However, it is useful to diagnose completely intrahepatic gallbladder. MRCP is considered to be the diagnostic method of choice. It is a noninvasive imaging technique that provides a detailed evaluation of the biliary tract [14], makes the preoperative and postoperative diagnosis of GA and excludes the condition of ectopic gallbladder too. According to the algorithm published by Male in 2010 [10], MRCP is the most appropriate imaging tool that should be used as the next investigation step when ultrasonography is not conclusive. In our case, the diagnosis was made in light of the radiological findings and the lack of a previous cholecystectomy. Due to its rarity, there are no specific guidelines for the management of GA. For symptomatic patients, most authors recommend conservative management based on the use of smooth muscle relaxants. Some others suggest sphincterotomy for severe cases [5]. In our case, the patient was asymptomatic, and no further treatment was needed. The patient was just informed about his congenital malformation, and we provided him with a copy of his medical record.

**Patient perspective**: the patient has been informed of his rare anatomical abnormality and has accepted the proposed treatment.

**Informed consent**: written informed consent was obtained from the patient for the publication of this case report and the accompanying images.

## Conclusion

Gallbladder is a rare congenital anomaly that should be kept in mind by radiologists and surgeons if the gallbladder is scleroatrophic or non-visualized on ultrasonography. Thus, further investigations should be performed. The correct preoperative diagnosis is mandatory to avoid unnecessary and dangerous surgery in symptomatic patients.
